# Whole genome sequencing identifies a novel species of the genus *Capnocytophaga* isolated from dog and cat bite wounds in humans

**DOI:** 10.1038/srep22919

**Published:** 2016-03-07

**Authors:** Salah Zangenah, Nasir Abbasi, Anders F. Andersson, Peter Bergman

**Affiliations:** 1 Department of Laboratory Medicine, Div of Clinical Microbiology, Karolinska Institutet and Karolinska University Hospital, Huddinge, Sweden; 2 KTH Royal Institute of Technology, Science for Life Laboratory, School of Biotechnology, Division of Gene Technology, Stockholm, Sweden; 3 Evolutionary Genomics Group, Departamento de Producción Vegetal y Microbiología,Universidad Miguel Hernández, San Juan de Alicante, 03550, Alicante, Spain

## Abstract

*C. canimorsus* and *C. cynodegmi* are dog and cat commensals which can be transmitted to humans via bites or scratches and can cause sepsis, meningitis, endocarditis, and eye- or wound infections. Recently an additional *Capnocytophaga* species was identified as part of the oral flora of healthy dogs and was given the name “C. canis”. We previously identified a *Capnocytophaga* isolate that could not be typed with available diagnostic tests including MALDI-TOF, 16S rRNA sequencing or species-specific PCR. This strain and 21 other *Capnocytophaga* spp isolated in Sweden from clinical blood- or wound-cultures were subjected to whole genome sequencing using the Illumina platform. Phylogenetic analysis revealed that the previously non-typable isolate belongs to the putative new species “C. canis”. Since this strain was isolated from a wound it also shows that members of “C. canis” have the potential to be pathogenic. In addition, our phylogenetic analysis uncovered an additional species of *Capnocytophaga*, which can be transmitted from dogs and cats to humans, suggesting a speciation within the *Capnocytophaga* family that has not been observed before. We propose the name of “C. stomatis” for this putative novel species.

The genus *Capnocytophaga* is comprised of eight known species. Six of them are part of the human oral flora: *C. gingivalis*, *C. granulosa*, *C. haemolytica*, *C. leadbetteri*, *C. ochracea*, and *C. sputigena* (formerly dysgonic fermenter-1 organisms). The two other members are zoonotic pathogens that can be found in the oral cavity of dogs and cats: *C. canimorsus* and *C. cynodegmi* (formerly ‘dysgonic fermenter-2′ and ‘dysgenic fermenter-2 like’ bacteria). In addition, a candidate novel species, “C. canis”, was recently identified in the oral flora of healthy dogs^1^. Except “C. canis”, All *Capnocytophaga* species are known to be medically important and can cause various types of infections ranging from minor wound infections to sepsis^2^,^3^,^4^. The human *Capnocytophaga* species are generally associated with oral diseases, such as periodontal infections, and are occasionally recovered from the respiratory tract^5^,^6^.

In this study we will focus on the animal associated *Capnocytophaga* spp, which can transmitted to humans via bites or scratches mainly from dogs or cats. In addition, the pine weevil was recently proposed as a possible vector for transmission of *C. canimorsus* to humans^7^. The best characterized of these species, *C. canimorsus*, has been reported to cause sepsis, meningitis and endocarditis, and has been described as a cause of minor wound infections. The other animal-associated *Capnocytophaga* species, *C. cynodegmi*, is considered to be less virulent and has mainly been associated with wound infections^8^.

*Capnocytophaga* are fastidious and slow growing bacteria, and diagnostic typing and characterization is difficult and normally takes several days. However, the introduction of MALDI-TOF in clinical bacteriology has significantly shortened the time to a correct diagnosis. In a previous project we compared the performance of traditional typing methods with MALDI-TOF and used 16S ribosomal RNA (rRNA) and species specific PCR as the reference method^8^. The main conclusion was that MALDI-TOF provided a rapid and reliable typing method in routine clinical bacteriology. Interestingly, in our previous study we identified one strain (W13), first isolated by us in 2007, which could not be identified to the species level using any of these methods^8^. Sequencing of the 16S rRNA gene and a subsequent BLAST-search resulted in 97% similarity with both *C. canimorsus* and *C. cynodegmi*^9^. To further elucidate the phylogenetic relationships in our collection of clinical and reference strains of *Capnocytophaga spp*, including the “non-typable” W13, all strains were subjected to whole genome sequencing. Here we present results suggesting that W13 belongs to the recently described novel species “C. canis”. In addition, we identify a group of three strains that form a sister clade to *C. cynodegmi* that represents a novel *Capnocytophaga* species.

## Results

### Phylogeny within the *Capnocytophaga* family

**Figure 1:**
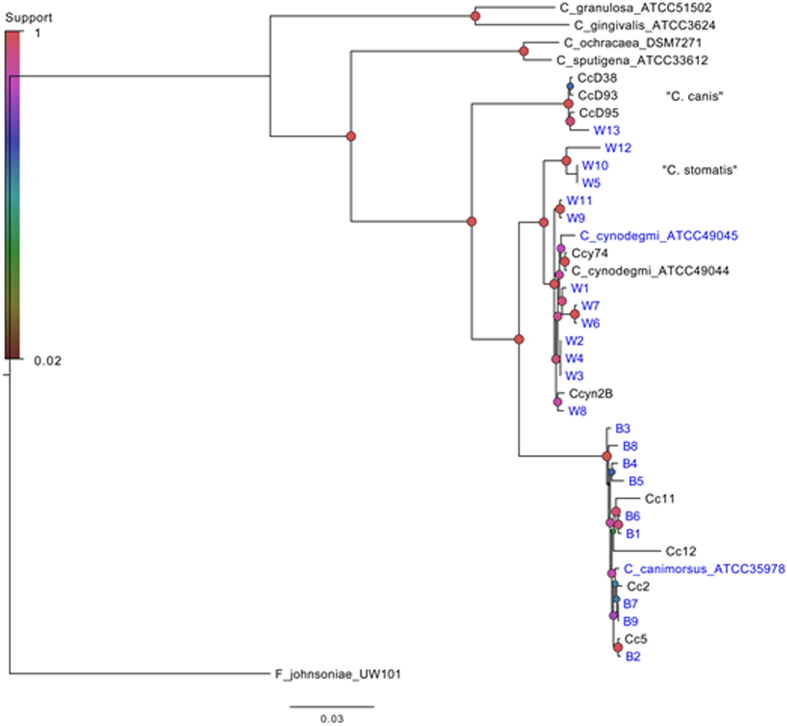
Phylogenetic analysis of *Capnocytophaga* strains. Maximum-likelihood trees inferred from concatenations of 43 housekeeping-gene proteins. *F. johnsoniae* UW101 was used as the outgroup. Strains isolated from blood and wounds are designated with “B” and “W”, respectively. CCD38, CCD93 and CCD95 and W13 represent the recently described species “C. canis”. W5, W10 and W12 represent the novel species, with the proposed name “C. stomatis”. Strains genome-sequenced in this study are indicated with blue text. The size and color of the circles indicate the reliability of the corresponding split in the tree using the Shimodaira-Hasegawa test (with 1 being the maximum possible value).

We previously attempted to determine the taxonomy of a collection of *Capnocytophaga* strains using 16S rRNA gene sequencing and species specific PCR. However, this was not successful for all strains and one strain (W13) could not be defined to the species level using these methods. Here we used whole genome sequencing to elucidate the phylogenetic relationships between the strains. After assembly, the draft genomes were supplemented with previously sequenced *Capnocytophaga* genomes and a phylogenetic tree was reconstructed using a concatenation of 43 core-gene proteins (Fig. 1). *Flavobacterium johnsoniae* was used as outgroup. Our isolate genomes clustered in three main clusters, with all blood isolates clustering around the *C. canimorsus* reference strains, and all wound isolates, except W13, clustering around the *C. cynodegmi* reference strains. Notably, strain W13 clustered together with the three recently published genomes of “C. canis” isolated from the oral flora of healthy dogs^1^. Three of the wound isolates (W5, W10 and W12) formed a sister clade to the *C. cynodegmi-*group. We also constructed phylogenetic trees using the 16S rRNA gene (data not shown) but found that there exists sequence variation between the different copies of the 16S gene within the genomes. For example, in *C. canimorsus* Cc5 the pairwise identity between the three copies was 98.9, 98.9 and 99.8%. Since different tree topologies were obtained with different copies of the 16S gene, we chose to use the concatenation of 43 core house-keeping genes for the phylogenetic analysis in this work (Fig. 1).

### Nucleotide similarity analysis

To further study the relationships within the *Capnocytophaga* family, an in silico DNA-DNA hybridization (DDH) approach was employed. Two prokaryotic organisms are typically regarded as different species if genomic DNA exhibits a DDH value <70%^10^,^11^. DDH predictions showed that W13 had low probability (0.07–5.07%) of displaying >70% DDH to the strains *C. canimorsus* and *C. cynodegmi* strains, but had a high DDH-score when compared to strains from the recently described “C. canis”-species (Table 1). Likewise, the strains W5, W10 and W12 had low probability (0.01–0.09%) of having >70% DDH to any of the strains outside their cluster (including *C. cynodegmi* strains). In contrast, the *C. canimorsus* and *C. cynodegmi* reference strains had high probabilities of displaying >70% DDH to the other strains within their respective clusters (Table 1).

### Gene-content analysis

**Figure 2:**
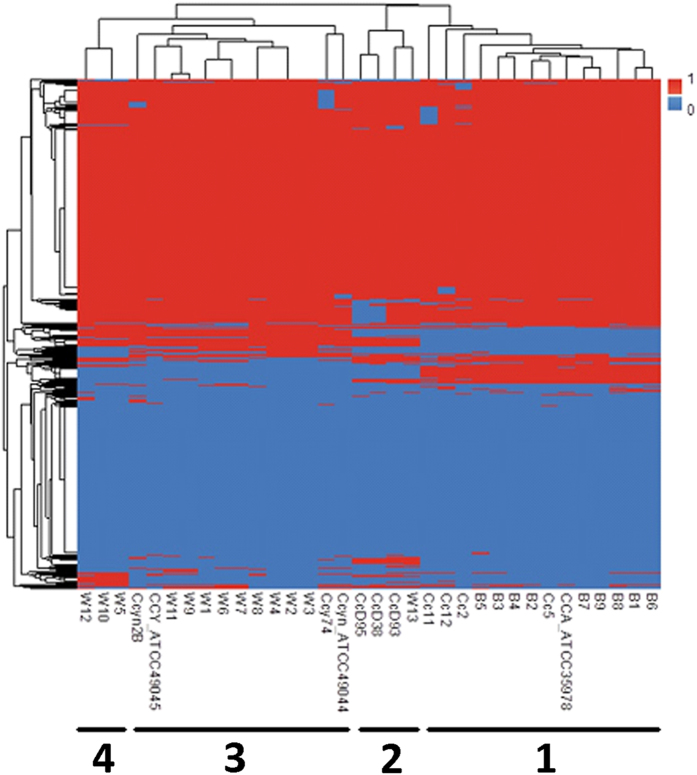
Heat-map representation of the presence (red = 1) or absence (blue = 0) of COGs in the different *Capnocytophaga* genomes. CCA, *C. canimorsus*, CCY, *C. cynodegmi* and Cc, “C. canis”.

**Figure 3:**
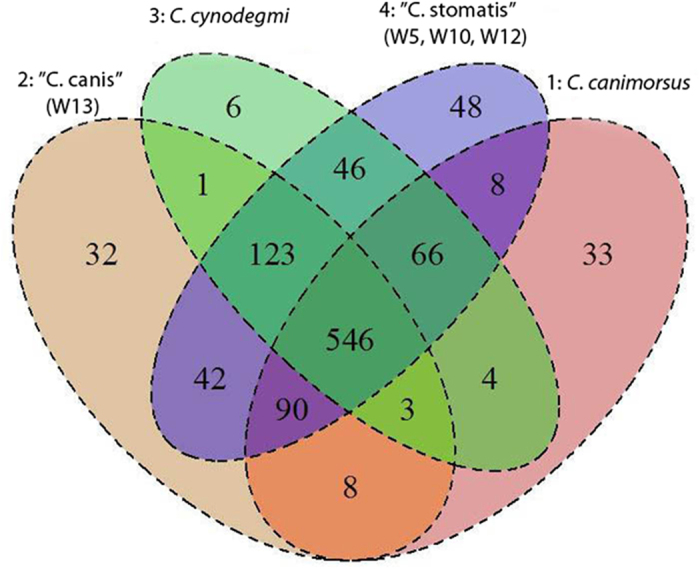
A Venn-diagram showing clusters of orthologous groups of genes (COGs) for the four clusters of strains. Cluster 1: *C. canimorsus* (pink); cluster 2: “C. canis”(isolates CCD38, CCD93, CCD95 and W13, beige); cluster 3: *C. cynodegmi* (green) and cluster 4: The novel species with the proposed name “C. stomatis” (isolates W5, W10 and W12, purple). Note that a COG was considered to be present in a cluster only if it was present in all genomes in the cluster.

In total 5451 clusters of orthologous groups of genes (COGs) were found among the 24 sequenced strains. A heat-map based on the pattern of presence/absence of the COGs gave further support to speciation in the *Capnocytophaga* genus (Fig. 2). The heat-map revealed four distinct clusters, with *C. canimorsus* strains to the far right (cluster 1), followed by “C. canis” (cluster 2) and *C. cynodegmi* (cluster 3) and the putative novel species to the far left (cluster 4). The gene content of the four clusters was further compared using a Venn-diagram (Fig. 3). The core genome consisted of 546 shared COGs, which were present in all strains from the four clusters (Fig. 3). The Venn-diagrams clearly showed that the recently described “C. canis” was different from the other clusters. Moreover, the novel cluster 4 (W5, W10 and W12), described by us, was also distinctly separated from the other clusters.

Several COGs were unique for the respective clusters (defined as being shared by all members of a cluster but not being shared by all members of any other cluster). For example, the genomes of *C. canimorsus* contained 33 unique COGs and seven of these were involved in inorganic ion transportation. “C. canis” had 32 unique COGs, of which four involved amino acid transport and metabolism, and three were involved in bacterial defense mechanisms. *C. cynodegmi* contained six unique COGs and three of these had functions in amino acid transport and metabolism. Finally, the novel species described here (W5, W10 and W12) had 48 unique COGs of which six involved coenzyme transport and metabolism, as well as biosynthesis of thiamine (Fig. 3).

### Phenotypic characterization of *Capnocytophaga*

**Figure 4:**
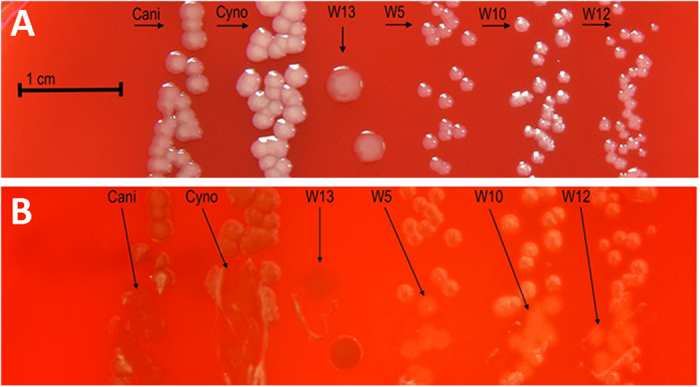
Colony morphology. (**A**) *C. canimorsus*, *C. cynodegmi*, W13, W5, W10 and W12 colony morphology on blood agar, 72 h culture, 37° C, 5% CO_2_ atmosphere. (**B**) Alpha hemolytic activity of *C. canimorsus*, *C. cynodegmi* and W13. Beta hemolytic activity of W5, W10 and W12 on blood agar, 72 h culture, 37° C, 5% CO_2_ atmosphere.

Like *C. canimorsus* and *C. cynodegmi*, the novel strain W13 and the strains W5, W10 and W12 were slow growing and capnophilic (requiring 5–10% CO_2_ atmosphere for optimal growth). The bacterial colony morphologies of the isolates on blood agar plates were similar. After 24 hours of incubation the colonies were mostly too small to be visible, but typical colonies were formed 48–72 h after incubation. Interestingly, the novel species differed from the reference strains with regard to size, color and shape. These differences became even more pronounced when colonies were examined after 72 hours of incubation (Fig. 4A). The reference strain of *C. canimorsus* had small (ca 1–2 mm), slightly raised transparent/greyish colonies on blood agar, whereas *C. cynodegmi* had a larger (2–3 mm), convex, translucent to opaque colony morphology. The colonies of strain W13 were flat, larger than *C. cynodegmi* and formed transparent/greyish colonies similar to those of *C. canimorsus* (Fig. 4A). Importantly, the colony morphologies of the novel strains W5, W10 and W12 were clearly distinct from the reference strains, with regards to size and shape (Fig. 4A,B). Strains W5, W10 and W12 exhibited beta hemolytic activity on blood agar (Table 2, Fig. 4B, arrows). Gram staining showed that the cell morphology was generally similar between the strains and typical for *Capnocytophaga* species, i.e. gram negative, slightly curved and with a fusiform shape (data not shown).

Finally, biochemical tests showed that W13 and the strains W5, W10 and W12 were clearly distinct from human *Capnocytophaga* species, due to positive reactions in oxidase, catalase and arginine dihydrolase tests, and exhibited a different biochemical reaction pattern (Table 2). All isolates showed positive reactions for hydrolysis of esculin and hydrolysis of orto-nitrophenol galactopyranoside (ONPG). Notably, W13 exhibited similar biochemical reactivity as both reference strains of *C. canimorsus* and *C. cynodegmi*, except for a negative reaction in the amygdalin test. The strains W5, W10 and W12 were similar to the reference strains with regard to most biochemical tests, but W5 and W10 were the only strains that showed positive result for raffinose.

## Discussion

### Summary of the data

Here we describe the phylogeny and gene content of a collection of *Capnocytophaga canimorsus* and *Capnocytophaga cynodegm*i isolates based on whole genome sequencing. The analysis also included a *Capnocytophaga* strain from a wound specimen (W13) that previously could not be defined as belonging to either *C. canimorsus* or *C. cynodegmi* by using available methods, including 16S rRNA sequencing or species specific PCR^8^. During the work of this project, a report was published where a large collection of *Capnocytophaga* strains from the normal oral flora of dogs were presented^4^. The authors of this report identified a putative novel *Capnocytophaga* species, named “C. canis”. The phylogenetic analysis presented here shows that our previously unidentified strain W13 belongs to “C. canis”.

We also identified three additional strains (W5, W10 and W12) that form a sister clade to *C. cynodegmi*, and we suggest that this group constitutes a novel *Capnocytophaga* species. We base these conclusions on several pieces of evidence: i) Phylogenetic analysis using 43 concatenated housekeeping-gene proteins placed the isolates W5, W10 and W12h as a sister group to *C. cynodegemi*; ii) DNA-DNA hybridization suggested that strains W5, W10 and W12 had low probability to have >70% similarity with other *Capnocytophaga* strains outside their own cluster. iii) Gene content analysis clustered W5, W10 and W12 together but outside the *C. cynodegemi* cluster. iv) Biochemical analyses suggested that strains W5, W10 and W12 differed compared to the reference strains, and W5, W10 and W12 exhibit hemolytic activity on blood agar plates.

### Relation to other studies

As discussed above, three additional *Capnocytophaga* genomes isolated from the oral flora of healthy dogs were recently published^12^. The authors of this recent study suggested that these commensal isolates could constitute a new “subgroup” of *C. canimorsus* by virtue of their genetic difference from clinical isolates associated with human infections. In a follow-up paper the same authors performed detailed analyses of these three strains and suggested that they are non-pathogenic due to exclusive association with dog normal flora, an inability to cleave glycan from human IgM and a lack of growth in heat-inactivated human serum^1^. Most “C. canis”-isolates were shown to be oxidase-negative in the paper from Renzi *et al.*, which is in contrast to *C. canimorsus* and *C. cynodegmi*. It is noteworthy that our strain, W13, was isolated from a human cat bite wound and is oxidase-positive, thus challenging the notion that “C. canis” is strictly a dog commensal. This could support a role for oxidase in the pathogenesis of *Capnocytophaga* species, which warrants further investigation.

### Implications for clinicians and scientists

Our finding of a potentially novel species of *Capnocytophaga*, which is associated with infections in humans, has important implications. First, we broaden the zoonotic landscape and expand the knowledge about bacterial species that can be transmitted to humans from cats and dogs. Second, our findings form a platform for further studies on the epidemiology, disease spectrum and prognosis for infections with these novel species. Finally, the access to whole genome information opens up new avenues for studies of bacterial physiology, antibiotic resistance and virulence traits associated with *Capnocytophaga*-infections, both with the known species *C. canimorsus* and *C. cynodegmi*, as well as with the novel species “C. canis”. In addition, the tentative novel species described by us deserves further attention, both in the clinical laboratory and in experimental science.

### Conclusions and future prospects

We present data suggesting that, in addition to *C. canimorsus, C. cynodegmi* and “C. canis” there is an additional novel species of *Capnocytophaga* that can be transmitted from cats and dogs to humans. We propose the name of “C. stomatis” for this species (from Greek ‘stoma’; mouth), comprising strains W5, W10 and W12. Increased awareness for *Capnocytophaga* in clinical laboratories will contribute to further understanding of the role that these novel *Capnocytophaga* species may have in various human infections.

## Materials and Methods

### Ethical statement

This work deals with clinical bacterial isolates from human infections. No tissue material or other biological material was obtained from humans. The Swedish law does not require ethical approval for work with bacterial isolates from humans. All information regarding these isolates is anonymized and is thus exempt from Swedish ethical approval. All experimental protocols were carried out in accordance with the approved guidelines for work with bacterial isolates at Karolinska Institutet, Stockholm, Sweden.

### Bacterial strains

All clinical isolates were collected in Sweden during the period 2005–2010. The blood-isolates were provided by Falun Central Hospital and Karolinska University Hospital, Sweden. All wound-isolates were identified at Karolinska University Hospital. Nine strains of *C. canimorsus* (blood isolates, B1–B9), 12 strains of *C. cynodegmi* (wound isolates, W1–12), one “non-typable” strain (wound isolate W13), and two reference strains ATCC 35978 (*C. canimorsus*, blood isolate) and ATCC 49045 (*C. cynodegmi*, wound isolate) were included in the study (n = 24 isolates in total).

The strain W13 was isolated from a cat bite wound culture of a 31 year old woman. Strain W5 was isolated from a cat bite wound culture of a 39 year old woman.W10 was isolated from a dog bite wound culture of a 61 year old man, and W12 was isolated from a deep wound culture (type of wound was not specified) of a 37 year old woman. Details on the other strains used here can be found in^8^.

### DNA extraction

DNA from the bacterial isolates was extracted using Thermo Scientific GeneJET Genomic DNA Purification Kit according to the manufacturer’s instructions for Gram-Negative Bacteria Genomic DNA Purification Protocol.

### Sequencing

Illumina TrueSeq libraries were prepared and sequenced on Illumina HiSeq instruments at the National Genomics Infrastructure at Science for Life Laboratory, Solna, Sweden. 24 isolates were sequenced on 2 HiSeq lanes with 500 x coverage per genome.

### Assembly

Sequence data for each isolate was down sampled to approximately 200 x coverage (estimated based on genome sizes of previously sequenced *Capnocytophaga*). Assembly was conducted with Velvet (20211242) using a kmer size of 83. Draft genome sequences have been deposited in the European Nucleotide Archive (ENA).

### Phylogenetic analysis

Contigs >1000 bp for each isolate sequenced here, as well as genomes (draft or complete) from *C. canimorsus* Cc12*, C. canimorsus* Cc2, *C. canimorsus* Cc5, *C. canimorsus* Cc11*, C. cynodegmi* Ccy74, *C. cynodegmi* ATCC49044*, C. cynodegmi* Ccyn2B, “C. canis” CcD38, “C. canis” CcD93, “C. canis” CcD95, *C. gingivalis* ATCC3624, *C. granulosa* ATCC51502, *C. ochracaea* DSM7271, *C. sputigena* ATCC33612 and *Flavobacterium johnsoniae* UW101 were analyzed with CheckM^13^ in order to automatically extract, concatenate and align the proteins of a set of 43 phylogenetically informative marker genes consisting primarily of ribosomal proteins and RNA polymerase domains. A phylogenetic tree was inferred from the concatenated alignment with FastTree^14^ under the WAG + GAMMA model and rooted between *F. johnsoniae* and the *Capnocytophaga* genomes. The reliability of each split in the tree was estimated using the Shimodaira-Hasegawa test in FastTree. FigTree (http://tree.bio.ed.ac.uk/software/figtree/) was used for tree visualizations. CheckM was also used to extract (full or partial) 16S rRNA genes from the genomes. These genes were further aligned with the Silva aligner^15^ and phylogenetic trees inferred from the alignment with FastTree.

### Annotation

Contigs >1000 bp for each isolate, as well as the other genomes used in the phylogenetic analysis, were annotated using PROKKA^16^, but supplemented with COG annotation for as many genes as possible through RPS-BLAST searches against NCBI’s conserved domain database^17^ using a E-value threshold of 10^−5^.

### COG analysis

A table with presence/absence of COGs in the different genomes was plotted with the pheatmap package in *R*^18^ using euclidean distances for clustering of rows (COGs) and columns (genomes). The VennDiagram package in *R* was used for generation of Venn-diagrams.

### Nucleotide similarity analysis

In silico genome DNA-DNA hybridization predictions using the Genome Blast Distance Phylogeny method were carried out using the online Genome-to-Genome Distance Calculator^10^. This online tool also reported probabilities for that a pair of genomes would yield a wet-lab DNA-DNA hybridization (DDH) value above 70%.

### Biochemical characterization

API 20e (bioMérieux) and classical biochemical test tubes (obtained from the Substrate Unit, Karolinska University Hospital, Huddinge, Stockholm, Sweden and described in Table 2) were used to determine biochemical characteristics of the bacterial strains.

## Additional Information

**How to cite this article**: Zangenah, S. *et al.* Whole genome sequencing identifies a novel species of the genus *Capnocytophaga* isolated from dog and cat bite wounds in humans. *Sci. Rep.* **6**, 22919; doi: 10.1038/srep22919 (2016).

## Figures and Tables

**Table 1:** Predicted genomic DNA-DNA hybridization (DDH) similarity between selected *Capnocytophaga* strains.

	CCA		CCY		W5		W10		W12		W13	
	DDH	Prob. DDH> = 70%	DDH	Prob. DDH> = 70%	DDH	Prob. DDH> = 70%	DDH	Prob. DDH> = 70%	DDH	Prob. DDH> = 70%	DDH	Prob. DDH> = 70%
CCA	100	98.3	44.1	6.89	29.4	0.08	29.4	0.08	29.6	0.09	42.6	5.02
B1	80.7	91.1	42.2	4.59	29.1	0.07	29.1	0.07	29.3	0.08	40.7	3.2
B2	84.4	93.3	43.7	6.36	29	0.07	29	0.07	29.3	0.08	42.5	4.95
B3	81.5	91.6	42.7	5.13	28.6	0.06	28.5	0.06	28.5	0.06	41.4	3.85
B4	79.7	90.3	43.2	5.74	28.6	0.06	28.6	0.06	29.4	0.08	43	5.41
B5	80.7	91.1	42.7	5.06	29	0.07	29	0.07	29	0.07	41.5	3.92
B6	80.8	91.1	41.9	4.26	29	0.07	29	0.07	28.9	0.06	41.1	3.59
B7	84.2	93.3	45.4	8.82	29.3	0.08	29.3	0.08	29.5	0.09	42	4.36
B8	79.5	90.1	43.2	5.7	28.7	0.06	28.7	0.06	28.8	0.06	41.8	4.2
B9	84.4	93.4	45.2	8.62	29.7	0.09	29.7	0.09	29.9	0.1	42	4.41
CCY	44.1	6.89	100	98.3	27.2	0.03	27.2	0.03	27.1	0.03	33.4	0.39
W1	44	6.67	69.7	77.7	27.5	0.03	27.5	0.03	27.6	0.04	34.4	0.54
W2	45.8	9.49	70.9	79.9	27.9	0.04	27.9	0.04	27.4	0.03	34.9	0.64
W3	45.8	9.48	70.9	79.9	27.9	0.04	27.9	0.04	27.4	0.03	34.9	0.64
W4	45.8	9.48	70.9	79.9	27.9	0.04	27.9	0.04	27.4	0.03	34.9	0.64
W5	29.4	0.08	27.2	0.03	100	98.3	100	98.3	74.9	85.6	28.2	0.05
W6	44.6	7.6	69.5	77.3	27.5	0.03	27.5	0.03	27.5	0.03	34.3	0.53
W7	44.6	7.61	69.5	77.3	27.5	0.03	27.5	0.03	27.5	0.03	34.3	0.53
W8	45.4	8.87	71.2	80.4	28	0.04	28	0.04	27.6	0.04	34	0.47
W9	44.7	7.75	69	76.4	27.6	0.04	27.6	0.04	27.5	0.03	32.6	0.29
W10	29.4	0.08	27.2	0.03	100	98.3	100	98.3	74.9	85.6	28.2	0.05
W11	44.7	7.74	69	76.5	27.6	0.04	27.6	0.04	27.5	0.03	32.6	0.29
W12	29.6	0.09	27.1	0.03	74.9	85.6	74.9	85.6	100	98.3	29.1	0.07
W13	42.6	5.02	33.4	0.39	28.2	0.05	28.2	0.05	29.1	0.07	100	98.3
Cc2	83.6	92.9	45.4	8.79	29.6	0.09	29.6	0.09	29.7	0.09	42.8	5.19
Cc5	83.2	92.7	43.4	5.93	28.7	0.06	28.7	0.06	29.3	0.08	42.4	4.82
Cc11	79.7	90.3	43.2	5.72	29.4	0.08	29.4	0.08	28.9	0.07	41.1	3.56
Cc12	80.6	91.0	43.1	5.58	28.5	0.05	28.5	0.05	28.8	0.06	39.4	2.35
CcD38	46.4	10.6	37.2	1.26	28.7	0.06	28.7	0.06	27.7	0.04	80.3	90.8
CcD93	45.9	9.66	36.6	1.07	28.0	0.04	28.0	0.04	28.5	0.05	80.3	90.7
CcD95	46.2	10.3	36.8	1.14	28.5	0.05	28.5	0.05	27.7	0.04	79.2	89.9
Ccy 74	42.7	5.12	69.8	78	28.1	0.04	28.1	0.04	27.8	0.04	33.4	0.39
Ccy4904	43.7	6.34	69.8	77.9	28.0	0.04	28.0	0.04	27.7	0.04	34.0	0.48
Ccyn2B	45.2	8.56	71.4	80.6	27.7	0.04	27.7	0.04	27.5	0.03	33.7	0.42

Predicted DDH levels (left columns) and probabilities (in percentage) for a DDH-similarity exceeding 70% are given (right columns) for each comparison. CCA, *C. canimorsus* and CCY, *C. cynodegmi.* CCD38, CCD93 and CCD95 and W13 represent the recently described species “C. canis” (yellow). W5, W10 and W12 represent the novel species described here with the proposed name “C. stomatis” (blue). Green highlight represents relevant comparisons described in the text.

**Table 2:** Biochemical reactions of *Capnocytophaga* spp.

Characteristic Reaction	Data from literature			ATCC Strains		“C. canis”	Putative novel species		
	Cani	Cyno	Human C. spp	Cani	Cyno	W13	W5	W10	W12
Hemolysis, SBA	**−**	**−**	**−**	**α**	**α/β**	**α**	**β**	**β**	**β**
Oxidase	**+**	**+**	**−**	**+**	**+**	**+**	**+**	**+**	**+**
Catalase	**+**	**+**	**−**	**+**	**+**	**+**	**+**	**+**	**+**
Indole	**−**	**−**	**−**	**−**	**−**	**−**	**−**	**−**	**−**
Arginine hydrolase	**+**	**+**	**−**	**+**	**+**	**+**	**+**	**+**	**+**
Esculin hydrolysis	**v**	**+**	**v**	**+**	**+**	**+**	**+**	**+**	**+**
ONPG hydrolysis	**+**	**+**	**v**	**+**	**+**	**+**	**+**	**+**	**+**
Amygdalin	**+/−**	**ND**	**ND**	**+**	**+**	**−**	**+**	**+**	**+**
Glucose	**+**	**+**	**+**	**+**	**+**	**w**	**+**	**+**	**+**
Lactose	**+**	**+**	**v**	**+**	**+**	**+**	**+**	**+**	**+**
Mannose	**v**	**+**	**+**	**−**	**−**	**−**	**−**	**−**	**−**
Melibiose	**+**	**+**	**−**	**−**	**+**	**−**	**+**	**+**	**+**
Raffinose	**−**	**+**	**v**	**−**	**−**	**−**	**+**	**+**	**−**
Sucrose	**−**	**+**	**+**	**−**	**−**	**−**	**+**	**+**	**+**

Minus-sign ‘−’, negative, Plus-sign ‘+’, positive; ND, no data available; v, variable; w, weak; Cani, *C. canimorsus*; Cyno, *C. cynodegmi.*
